# Circ0038632 modulates MiR-186/DNMT3A axis to promote proliferation and metastasis in osteosarcoma

**DOI:** 10.3389/fonc.2022.939994

**Published:** 2022-08-18

**Authors:** Xinyu Tan, Canjun Zeng, Haomiao Li, Yeru Tan, Hongbo Zhu

**Affiliations:** ^1^ The Third Affiliated Hospital of Southern Medical University, Guangzhou, China; ^2^ Department of Medical Oncology, The First Affiliated Hospital, Hengyang Medical School, University of South China, Hengyang, China

**Keywords:** circ0038632, miR-186, DNMT3A, proliferation, metastasis, osteosarcoma

## Abstract

Osteosarcoma is a highly malignant solid tumor with poor prognosis, early metastasis, and rapid progression and has a high mortality rate, in which better therapeutic strategies are needed. Circ0038632, also known as circPLK1, is a tumor promotor in multiple cancers. However, its biological functions and molecular regulatory mechanisms in osteosarcoma remain unclear. To ascertain the function of circ0038632 in osteosarcoma, we checked its expression in cells and in tissues and tested its abilities of proliferation and migration. Expression experiment manifested that circ0038632 showed an enhanced expression in osteosarcoma. Functional studies revealed that circ0038632 inhibition reduced cell proliferation and metastasis abilities of osteosarcoma. Mechanism studies revealed that circ0038632 sponged miR-186 to upregulate the expression of DNA methyltransferase 3A (DNMT3A) to promote osteosarcoma progression. The circ0038632/miR-186/DNMT3A axis was involved in osteosarcoma progression. The results elucidated the potential application of circ0038632 as a novel diagnostic biomarker for progressive process of osteosarcoma.

## Introduction

Osteosarcoma, one of highly malignant solid tumors, is originated from the bone marrow cavity. It usually occurs in adolescents aged 10–20 years, with a high degree of malignancy, poor prognosis, early metastasis, and rapid progression and a high mortality rate (1). Unfortunately, the cause, progression, and their mechanisms have not been clearly clarified up to now.

Circular RNAs (circRNAs) perform important function in tumor-related gene expression regulating and tumor progression. Recently, increasing studies revealed that circRNAs were deeply involved in malignancy (2). CircRNA_103801 induces osteosarcoma proliferation, migration, and invasion processes by mediating miR-338-3p (3). CircTADA2A accelerates the malignant progression of osteosarcoma *via* modulating miR-203a-3p (4). All these studies reveal that circRNAs exert important functions in osteosarcoma.

Circ0038632, also known as circPLK1, is a tumor promotor in multiple cancers. In breast cancer, circPLK1 has high-level expression and is closely related with poor survival, which may serve as the prognostic biomarker or therapeutic target (5). By mediating miR-1294/HMGA1, circPLK1 promotes the progression of non–small cell lung cancer (6). However, circ0038632 function and its inner regulatory mechanisms in osteosarcoma are still unclear. Therefore, elucidation the role of circ0038632 involving occurrence or progression in osteosarcoma is significant.

Here, we found that circ0038632 showed a boosted expression both in osteosarcoma cells and in tissues. Circ0038632 silencing decreased the proliferation and metastasis abilities of osteosarcoma cells. The process may implement by circ0038632 sponging miR-186, followed by increasing the expression of DNA methyltransferase 3A (DNMT3A), and finally promoting osteosarcoma progression. Aberrant regulation of DNMT3A is important to the tumorigenesis of multiple cancers, which was also confirmed in osteosarcoma (7, 8). Circ0038632 has a potential to function as diagnostic biomarker or therapeutic target in osteosarcoma treatment.

## Results

### circ0038632 is upregulated in osteosarcoma

**Figure 1:**
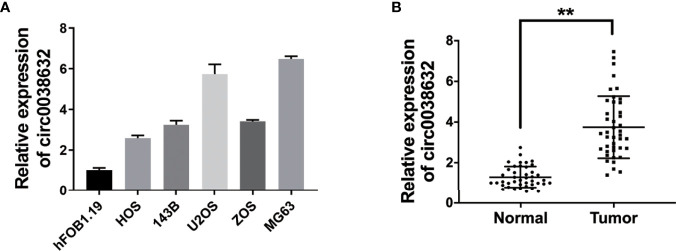
circ0038632 is upregulated in osteosarcoma. **(A)** The circ0038632 expression in osteosarcoma cell lines. **(B)** The circ0038632 expression in 42 pairs of osteosarcoma tissues and adjacent normal tissues. **P < 0.01.

Relative mRNA expression of circ0038632 was monitored through quantitative real-time PCR (qRT-PCR), and it was found that it is markedly upregulated in osteosarcoma cell lines in comparison with its control cell (**Figure 1A**). Next, circ0038632 expression levels were verified in 42 pairs of osteosarcoma tissues and their corresponding paracancerous controls. The results demonstrated that circ0038632 had an enhanced expression in osteosarcoma tissues compared with their control tissues (**Figure 1B**).

### circ0038632 silencing suppresses the function of proliferation and metastasis in osteosarcoma

**Figure 2:**
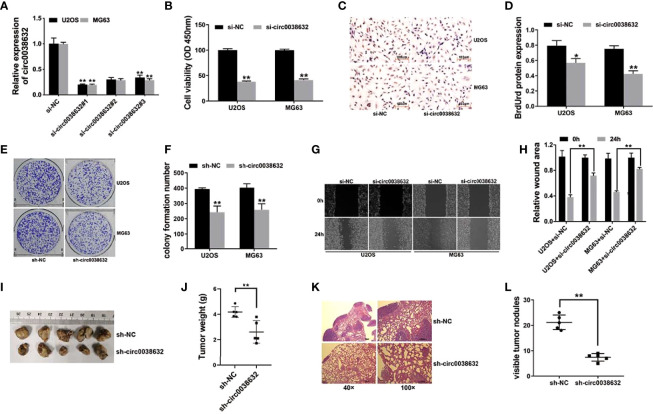
circ0038632 silencing suppresses the function of proliferation and metastasis in osteosarcoma. **(A)** siRNAs were used to knockdown circ0038632 expression in osteosarcoma cells. **(B)** Cell proliferation ability was evaluated through CCK-8 assay. **(C)** BrdUrd incorporation was assessed by immunohistochemistry (IHC) to evaluate cell proliferation after transfection. **(D)** Statistical chart of BrdUrd total gray value. **(E)** The pictures of clone formation from osteosarcoma cells were showed. **(F)** Statistical chart of cloning derived from osteosarcoma cells. **(G)** Migration ability was assessed by wound healing assay. **(H)** Statistical chart of wound closure. **(I)** Mouse xenograft models were established to evaluate circ0038632 intravital function. **(J)** Statistical chart of tumor weights. **(K)** The pictures of lung metastatic nodules with HE staining were showed. **(L)** Statistical chart of metastatic nodules. *P < 0.05 and **P < 0.01.

siRNAs were used to knockdown circ0038632, and si-circ0038632#1 was chosen for the following experiments (**Figure 2A**). Cell counting kit-8 (CCK-8) and BrdUrd assays were used to find that circ0038632 silencing restrained the proliferation ability of osteosarcoma cells (**Figures 2B**–**D**). Moreover, circ0038632 silencing depressed the colony formation capability of osteosarcoma cells (**Figures 2E**, **F**). In addition, circ0038632 silencing decreased the cell migration ability in osteosarcoma (**Figures 2G**, **H**). Finally, we used mouse xenograft models to investigate circ0038632 function *in vivo*. The results turned out that inhibition of circ0038632 suppressed the capability of growth (**Figures 2I**, **J**) and lung metastasis (**Figures 2K**, **L**) of osteosarcoma.

### circ0038632 serves as miR-186 sponge in osteosarcoma

**Figure 3:**
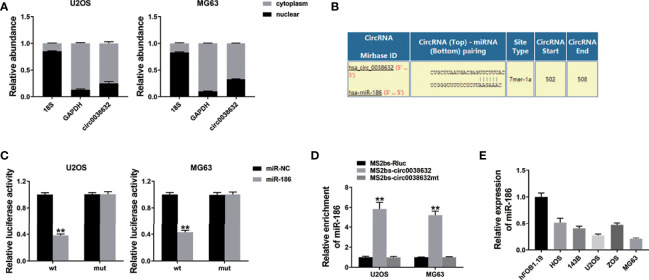
circ0038632 serves as miR-186 sponge in osteosarcoma. **(A)** qRT-PCR detected circ0038632, cytoplasmic control (GAPDH), and nuclear control (18S) expression. **(B)** Potential binding sites between miR-186 and circ0038632. **(C)** Relative luciferase activities of different co-transfected groups. **(D)** The enrichment of MS2bs-circ0038632, MS2bs-circ0038632mt, or control group was assessed. **(E)** The expression of miR-186 in osteosarcoma cell lines. **P < 0.01.

circRNAs could regulate gene expression by acting as microRNA decoys, also known as the competing endogenous RNA (ceRNA) mechanism. In osteosarcoma cells, cellular location of circ0038632 was inspected and found that circ0038632 mainly localized in cytoplasm (**Figure 3A**). Next, the prediction of potential binding sites between circ0038632 and miR-186 was accomplished adopting the online software Interactom (**Figure 3B**). Then, wild-type and mutant sequences of the binding sites between miR-186 and circ0038632 were designed, and we found that miR-186 + WT 3′-circ0038632 co-transfection group expressed a strikingly inhibited relative luciferase activity in osteosarcoma cells, when compared with miR-186 + MUT 3′-circ0038632 co-transfection group. In addition, RNA immunoprecipitation (RIP) assay was adopted and the results displayed that miR-186 enrichment was markedly increased in MS2bs-circ0038632 group (**Figure 3D**), showing that circ0038632 could serve as miR-186 sponge in osteosarcoma. Moreover, miR-186 manifested a tendency of distinct decrease in osteosarcoma cells compared with their control cells (**Figure 3E**).

### circ0038632 functions as a ceRNA by mediating DNMT3A in osteosarcoma

**Figure 4:**
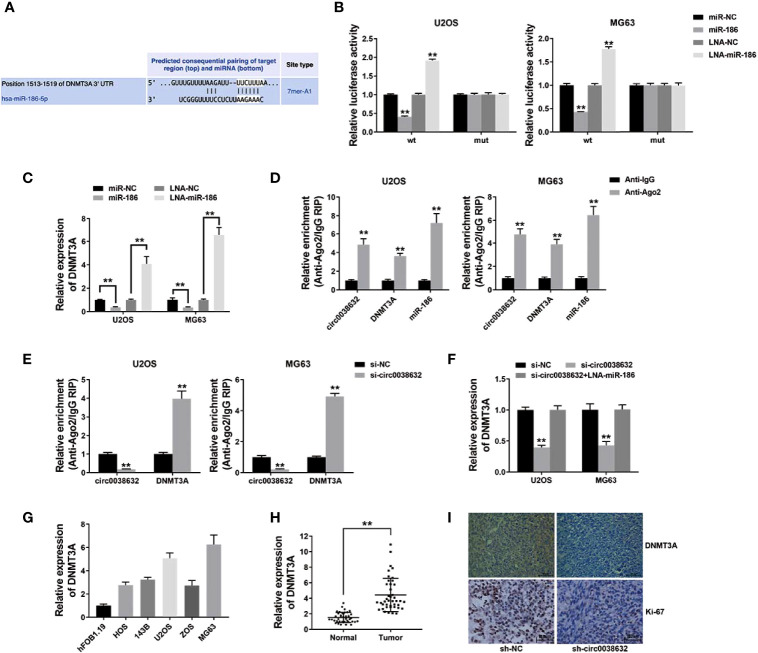
circ0038632 functions as a ceRNA by mediating DNMT3A in osteosarcoma. **(A)** Potential binding sites from 3′-UTR of DNMT3A with miR-186. **(B)** Relative luciferase activities of different co-transfected groups. **(C)** DNMT3A expression was tested after transfection by qRT-PCR. **(D)** The degree of circ0038632, DNMT3A, and miR-186 enrichment to Ago2 was examined by RIP assay. **(E)** The enrichment on Ago2 after transfection was tested. **(F)** DNMT3A mRNA expression was examined. **(G)** DNMT3A mRNA expression in osteosarcoma cells was tested. **(H)** DNMT3A mRNA expression in osteosarcoma tissues and its control were detected. **(I)** IHC staining of DNMT3A and Ki-67 were shown. **P < 0.01.

The prediction of potential downstream target genes of miR-186 was performed using the online software TargetScan, and we found that DNMT3A was among them (**Figure 4A**). Wild-type and mutant sequences were designed for the 3′-UTR of DNMT3A. The results showed that DNMT3A WT 3′-UTR + miR-186 co-transfection group showed a sharp decrease relative luciferase activity in osteosarcoma, and DNMT3A WT 3′-UTR + miR-186 inhibitor co-transfection group showed an apparent increase compared with DNMT3A MUT 3′-UTR + miR-186 co-transfection group (**Figure 4B**). Moreover, miR-186 was conductive to the inhibition of DNMT3A expression, and miR-186 inhibitor reversed DNMT3A expression (**Figure 4C**), revealing the expression that its target gene DNMT3A could be regulated by miR-186 in osteosarcoma.

Subsequent RIP assay results demonstrated that circ0038632, DNMT3A, and miR-186 enrichment was majorly raised in anti-AGO2 group in osteosarcoma cells (**Figure 4D**). Circ0038632 silencing significantly raised the enrichment of DNMT3A to Ago2 (**Figure 4E**), suggesting that circ0038632 and DNMT3A were rivalrous to binding with miR-186. Moreover, inhibition of circ0038632 decreased the expression of DNMT3A, but miR-186 inhibitor could rescue the decrease (**Figure 4F**), revealing that circ0038632 could sponge miR-186 and regulate the expression of DNMT3A in osteosarcoma. Next, DNMT3A expression was checked in osteosarcoma cells and tissues, and we found it had a noticeable raised trend (**Figures 4G**, **H**). DNMT3A expression in circ0038632 silencing group from mouse xenograft models showed a markedly reduction (**Figure 4I**). In addition, circ0038632 knockdown also decreased the expression of Ki-67 in the xenograft tumors (**Figure 4I**).

## Discussion

Although neoadjuvant chemotherapy combined with surgery has improved the survival probability of osteosarcoma in early-stage recently, current reports show that the 5-year patient survival of metastatic osteosarcoma can only reach 30% (9). Moreover, radical surgical treatment of osteosarcoma often leads to severe functional impairment and even disability of the patient’s limbs. Therefore, it is urgent to explore new treatment methods for osteosarcoma.

circRNAs play critical roles in cancer progression. Circ0038632 is a progression promotor in multiple malignant tumors. By mediating miR-1294/HMGA1, circPLK1 promoted the abilities of malignant pleural mesothelioma to proliferate, migrate, invade, and keep stemness (10). CircPLK1 silencing inhibited the growth, migration, and invasion of breast cancer (11). However, how circ0038632 functions in osteosarcoma has not yet been revealed. Here, we results revealed that circ0038632 had an enhanced expression in both osteosarcoma cells and tissues (**Figure 1**). In addition, knockdown of circ0038632 suppressed the capability of proliferation and metastasis of osteosarcoma cells (**Figure 2**).

Next, we investigated the mechanism of circ0038632 function in osteosarcoma. We explored the potential circRNA/miRNA interaction for circ0038632 and miR-186. MiR-186 is a candidate of tumor suppressors in multiple cancers. In osteosarcoma, miR-186-5p has been proved to downregulate and negatively relate to survival (12). Overexpressed miR-186 restrained the proliferation, invasion, and aerobic glycolysis process in osteosarcoma (13). Moreover, miR-186-5p repressed osteosarcoma migration or invasion through regulating TBL1XR1 expression (14). In this study, miR-186 manifested a tendency of distinct decrease in osteosarcoma cells (**Figure 3**).

The ceRNA mechanism involved miR-186 was reported in several studies. lncRNA DSCAM-AS1 promotes malignant transformation in osteosarcoma by sponging miR-186-5p (15). lncRNA NEAT1 sponges miR‐186‐5p and promotes the progression of osteosarcoma invasion and EMT (16). *Via* miR-186-5p, circ_0001174 promotes osteosarcoma progression (17). Our results showed that circ0038632 could regulate the expression of miR-186 by sponging it (**Figure 3**).

Aberrant regulation of DNMT3A is important to the tumorigenesis of multiple cancers, which has the potential to be a therapeutic target (7). DNMT3A is overexpressed and closely related with worse survival in pancreatic cancer (18). DNMT3A induces promoter methylation and miR‐200b silencing to promote tumor progression in breast cancer (19). In osteosarcoma, DNMT3A promotes APCDD1 promoter DNA hyper-methylation, downregulates its expression, and promotes cell invasion and metastasis (8). Here, DNMT3A expression was found to be noticeably increased in osteosarcoma (**Figure 4**).

The ceRNA mechanisms that involved DNMT3A were also reported in several studies. In breast cancer, circIQCH sponges miR-145 to accelerate proliferation and migration process through upregulating DNMT3A (20). Circ_0084615 could regulate the expression levels of DNMT3A be mediating miR-599 (21). Here, we found that circ0038632 could sponge miR-186 followed by regulating its downstream target gene DNMT3A (**Figure 4**). circRNAs are promising biomarkers for diagnosis and targets for therapeutic management (22). Our study confirmed the cancer-promoting effect of circ0038632 in osteosarcoma, which may lead to the identification as a potential disease biomarker or novel therapeutic target.

In conclusion, we showed that the circ0038632/miR-186/DNMT3A axis was involved in osteosarcoma proliferation and metastasis. Targeting circ0038632 has the potential to be a specific therapeutic strategy for osteosarcoma.

## Materials and methods

### Cell culture and transfection

Human osteosarcoma cells including U2OS, ZOS, 143B, MG63, HOS, and their control cells, osteoblast hFOB1.19, were obtained from ATCC. DNA fingerprinting was used for cell identification. The detection for mycoplasma infection was performed routinely.

Lipofectamine 3000 was adopted for cell transfection (Invitrogen, USA). siRNAs and shRNAs of circ0038632 and mimics and inhibitors of miR-186 were purchased from GeneCopoeia (USA). The sequences of siRNAs used in this study are as follows: si-NC, UUCUCCGAACGUGUCACGUTT; si-circ0038632#1, ACAGATGTGAATATTCTCTCT; si-circ0038632#2, GATGTGAATATTCTCTCTCCT; and si-circ0038632#3, GTGAATATTCTCTCTCCTGGA. The primer sequences for qRT-PCRs used in this study are as follows: circ0038632: forward (5′- 3′), TACATGTTCGGGTGTGGGTT; reverse (5′- 3′), CTTTCCTCCTCTTGTGCAGC; 18S: forward (5′- 3′), TTAATTCCGATAACGAACGAGA; reverse (5′- 3′), CGCTGAGCCAGTCAGTGTAG; GAPDH: forward (5′- 3′), GGAGCGAGATCCCTCCAAAAT, reverse (5′- 3′), GGCTGTTGTCATACTTCTCATGG; and DNMT3A: forward (5′- 3′), TATGAACAGGCCGTTGGCATC, reverse (5′-3′), AAGAGGTGGCGGATGACTGG.

### Quantitative real-time PCR analysis

TRIzol (Invitrogen), NE-PERTM Nuclear, and Cytoplasmic Extraction Reagents (Thermo, USA) were used to extract total RNA or nuclear and cytoplasmic RNA. PrimeScriptTM RT reagent Kit and TB Green Premix Ex TaqTM (RR037A, RR420A, Takara, Japan) were used for qRT-PCR assays. The primers were obtained from GeneCopoeia (Table S2).

### Clinical sample collection

Forty-two pairs of primary osteosarcoma samples and their corresponding paracancerous tissue samples were acquired from the Third Affiliated Hospital of Southern Medical University, immediately followed by keeping in liquid nitrogen. qRT-PCR was used to test the expression of circRNA. The research involving human samples and animals was reviewed and approved by the Ethics Committee of the Third Affiliated Hospital of Southern Medical University. All patients provided written informed consents. Declaration of Helsinki revised in 2013 was strictly implemented throughout the research.

### Cell counting kit-8 assay

After transfection, 10^3^ cells were resuspended and transferred into each well of 96-well plates. After 48 h of incubation at 37°C, 10 μl of CCK-8 solution was added. Then, absorbance of 450 nm was measured 2 h later.

### BrdUrd proliferation assay

BrdUrd proliferation assay was applied for cell viability detection. Cells were incubated under BrdUrd at 10 nmol/L for 16 h to test BrdUrd incorporation, followed by 1:1 cold methanol/acetone fixation. Then, immunocytochemistry detection was subsequently performed.

### Colony formation assay

After transfection, 10^3^ cells were resuspended and transferred into each well of six-well plates. After 14 days of incubation at 37°C. Then, methanol was used to fix the colonies, followed by crystal violet (0.1%) staining. The colonies were counted under the microscopy later.

### Wound healing assay

After transfection, 10^3^ cells were resuspended and transferred into each well of six-well plates. Pipette tips (10 μl) were used make the linear wound. Migration ability was assessed under microscope.

### Mouse xenograft model

The whole process of animal experiments was performed according to the guidelines of Institutional Animal Care and Use Committee of The Third Affiliated Hospital of Southern Medical University. A total of 10^6^ U2OS cells stably expressing sh-circ0038632 or its control were subcutaneously injected into each 4-week-old mouse (n = 5, male nude). Tumors were excised for following weights examine 28 days later.

A total of 10^5^ U2OS cells stably expressing sh-circ0038632 or its control were injected into each mouse through tail vein (n = 5). Lungs were excised for the following pathological studies 56 days later. The metastatic sites in lungs were counted under microscopy.

### Luciferase reporter assay

A total of 10^4^ cells were resuspended and transferred into each well of six-well plates. Luciferase-reporting plasmid containing wild or mutant sequences of circ0038632, DNMT3A 3′-UTR, miR-186 mimics, miR-186 inhibitors, and their controls were purchased. The mimics and inhibitors of miR-186 and the wild or mutant reporting vectors were co-transfected. The activities of renilla and firefly luciferase were assessed 48 h after transfection.

### RNA immunoprecipitation

MS2bs vector containing wild or mutant sequences of circ0038632 was constructed. The enrichment of circ0038632, miR-186, SLC7A11, and DNMT3A in osteosarcoma cells after transfection of the above vectors was detected by RIP assay using anti-Ago2 antibody, and qRT-PCR detection was completed after RNA purification.

### Immunohistochemistry analysis

After deparaffinization and rehydration by xylene, absolute ethanol, and 70% alcohol, slides were drained, citrate buffer was used for antigen retrieval, and PBS was used for washing, followed by 3% H_2_O_2_ and 5% goat treatment. Anti- DNMT3A (1:200, LSBio, USA) and specific secondary antibodies (1:500, Abcam, USA) were utilized to incubate the slides under the condition of 4°C overnight and room temperature for 2 h, respectively. DAB staining reagent (Beyotime, China) was utilized to stain the slides and then photographed under microscopy.

### Statistical analysis

All data were analyzed by SPSS 25.0 software and presented as mean ± SD. T-test was utilized for analyses of between difference groups. Statistical significance was set at p ≤ 0.05.

## Data availability statement

The raw data supporting the conclusions of this article will be made available by the authors, without undue reservation.

## Ethics statement

This study was reviewed and approved by Ethics Committee of the Third Affiliated Hospital of Southern Medical University. The patients/participants provided their written informed consent to participate in this study. Written informed consent was obtained from the owners for the participation of their animals in this study.

## Author contributions

All experiments were designed by XT and HZ. XT executed the experiments. CZ gathered specimens. HL and YT analysed and demonstrated the data. XT and HZ were the major contributors in writing and revising the manuscript. All authors read and approved the final manuscript. All authors contributed to the article and approved the submitted version.

## Funding

This study was supported by the Natural Science Foundation of Guandong (No. 2021A1515012095, Xinyu Tan).

## Conflict of interests

The reviewer (ZYD) declared a shared parent affiliation with the authors (XT, CZ, HL) to the handling editor at the time of review.

The authors declare that the research was conducted in the absence of any commercial or financial relationships that could be construed as a potential conflict of interest.

## Publisher’s note

All claims expressed in this article are solely those of the authors and do not necessarily represent those of their affiliated organizations, or those of the publisher, the editors and the reviewers. Any product that may be evaluated in this article, or claim that may be made by its manufacturer, is not guaranteed or endorsed by the publisher.
